# Evidence for hybridization and introgression within a species-rich oak (*Quercus *spp.) community

**DOI:** 10.1186/1471-2148-7-218

**Published:** 2007-11-10

**Authors:** Alexandru L Curtu, Oliver Gailing, Reiner Finkeldey

**Affiliations:** 1 Department of Forest Genetics and Forest Tree Breeding, Büsgen-Institute Georg – August University Göttingen, Büsgenweg 2, Göttingen, , 37077, Germany; 2 Department of Forest Sciences, Transilvania University Brasov, Sirul Beethoven 1, Brasov, 500123, Romania

## Abstract

**Background:**

Analysis of interspecific gene flow is crucial for the understanding of speciation processes and maintenance of species integrity. Oaks (genus *Quercus*, *Fagaceae*) are among the model species for the study of hybridization. Natural co-occurrence of four closely related oak species is a very rare case in the temperate forests of Europe. We used both morphological characters and genetic markers to characterize hybridization in a natural community situated in west-central Romania and which consists of *Quercus robur*, *Q. petraea*, *Q. pubescen*s, and *Q. frainetto*, respectively.

**Results:**

On the basis of pubescence and leaf morphological characters ~94% of the sampled individuals were assigned to pure species. Only 16 (~6%) individual trees exhibited intermediate morphologies or a combination of characters of different species. Four chloroplast DNA haplotypes were identified in the study area. The distribution of haplotypes within the white oak complex showed substantial differences among species. However, the most common haplotypes were present in all four species. Furthermore, based on a set of 7 isozyme and 6 microsatellite markers and using a Bayesian admixture analysis without any a priori information on morphology we found that four genetic clusters best fit the data. There was a very good correspondence of each species with one of the inferred genetic clusters. The estimated introgression level varied markedly between pairs of species ranging from 1.7% between *Q. robur *and *Q. frainetto *to 16.2% between *Q. pubescens *and *Q. frainetto*. Only nine individuals (3.4%) appeared to be first-generation hybrids.

**Conclusion:**

Our data indicate that natural hybridization has occurred at relatively low rates. The different levels of gene flow among species might be explained by differences in flowering time and spatial position within the stand. In addition, a partial congruence between phenotypically and genetically intermediate individuals was found, suggesting that intermediate appearance does not necessarily mean hybridization. However, it appears that natural hybridization did not seriously affect the species identity in this area of sympatry.

## Background

Natural hybridization and introgression can play an important role in evolution, e.g. by formation of new species or increasing genetic variation within species [1-3]. Hybridization is a quite common phenomenon in many organismal groups, particularly in plants [4]. However, the occurrence of natural hybridization is not universal, but concentrated in a small fraction of plant families and genera [4]. A well-known example is the genus *Quercus *(the oaks), in which many species are known to hybridize [5]. Because of their propensity to hybridize the biological species concept based largely on effective genetic isolation can not be applied to *Quercus *[6]. To overcome the problems with the reproductive species concept, another concept that relies on ecology was proposed with reference to oaks [7].

The occurrence of rare natural forms with intermediate morphologies was often interpreted as the result of a hybridization event. However, within oak species, morphology alone does not allow to detect putative hybrids, since the parental species are not sufficiently distinct and possess a wide variability [5]. For instance, no single morphological feature can unambiguously distinguish *Q. robur *from *Q. petraea*. However, it is possible to differentiate two distinct groups and thus to identify intermediate forms by using various multivariate analyses [e.g., [8,9]]. Furthermore, defining the limits between the 'typical' and 'intermediate' individuals is often more or less arbitrary [9]. Morphologically intermediate forms which are suspected to be hybrids are regularly observed in natural mixed populations (e.g., [10,11]).

Various types of genetic markers have been applied in studies on hybridization of oaks. Chloroplast DNA studies revealed that the most frequent chloroplast DNA variants are shared among related oak species which was interpreted as evidence for hybridization and introgression between taxa (e.g., [12-14]). Hybridization as a mechanism of invasion by one species (*Q. petraea*) into the range occupied by another species (*Q. robur*) through pollen swamping was proposed to account for the lack of differentiation between these two species [15,16]. However, a relatively low level of hybridization between *Q. robur *and *Q. petraea *was detected by paternity analysis in a mixed stand [17]. Studies of hybrid zones using morphology and molecular markers reported moderate levels of gene flow between different oak species (e.g., [11,18]). Recently, another study [19] suggested that low differentiation between *Q. robur *and *Q. petraea *results from shared ancestral variation rather than high rates of gene flow.

So far, most of the studies dealing with hybridization in oaks were carried out in mixed stands consisting of two species (e.g., *Q. robur *and *Q. petraea*). However, the higher number of sympatric oak species that coexist naturally at different sites in the eastern part of the European continent (e.g., North Balkan) provides new opportunities to investigate processes driving speciation, such as hybridization and introgression. Here, we examine a natural community of four closely related oak species. In a companion paper [20] the genetic variation and differentiation among the species present at this site is described in detail. The present paper will address the following questions: (i) does morphological grouping reflect the underlying genetic structure inferred from individual multilocus genotypes? (ii) is there any evidence of hybridization and introgression between species? (iii) is the level of introgression between species related to the non-random spatial distribution of oak species at this site?

## Results

### Morphological analysis

Based on the assessment of six characters we were able to identify 12 types of pubescence in the data set (Table 1). The most frequent types (1, 2 and 3, respectively), with a total frequency of 95.2%, correspond to those previously described in Central Europe ([21], p. 77). These types are taxa-specific and were used to discriminate between species. According to [21] a 'typical' *Q. robur *has neither stellate nor fasciculate hairs on the abaxial surface of the leaf – type 1 in our study; the most common type of *Q. petraea *has stellate hairs on the abaxial surface of the leaf and fasciculate hairs solely along the mid-rib – type 2; the 'typical' *Q. pubescens *has fasciculate hairs on the leaf, mid-rib, petiole and twig, whereas stellate hairs are missing – type 3 (Table 1). *Q. frainetto *can not be distinguished from *Q. pubescens *on the basis of pubescence alone. The individual trees that exhibit type 1, 2 and 3 were classified in this study as *Q. robur sensu stricto *(s.s.), *Q. petraea *s.s. and *Q. pubescens*-*Q frainetto *s.s., respectively.

**Table 1:** Species separation based on characters of pubescence

Character									
				
SH	FH	AMR	OMR	PET	TW	Type no.	Tree number	Taxon	No. of trees
0	0	0	0	0	0	1	-	*Q. robur *s.s.	65
1	0	1	0	0	0	2	-	*Q. petraea *s.s.	66
0	1	1	1	1	1	3	-	*Q. pubescens *– *Q. frainetto *s.s.	125
								Total – s.s.	256
0	0	1	0	0	0	4	68, 200	*Q. robur *– *Q. petraea *s.l.	2
1	0	0	0	0	0	5	3, 118		2
0	1	1	0	0	0	6	128	*Q. pubescens *– *Q. frainetto *s.l.	1
0	0	1	0	1	1	7	67		1
0	0	1	1	1	1	8	27		1
0	1	1	1	0	0	9	54		1
0	1	1	0	1	0	10	249		1
0	1	1	0	1	1	11	5, 24		2
0	1	1	1	1	0	12	124, 321		2
								Total – s.l.	13
									
Total									269

SH – stellate hairs on the abaxial surface of the leaf; FH – fasciculate hairs on the abaxial surface of the leaf; AMR – fasciculate hairs along the mid-rib; OMR – fasciculate hairs on the mid-rib; PET – fasciculate hairs on the petiole; TW – fasciculate hairs on the twig; 1 – present, 0 – absent; s.s. – *sensu stricto*; s.l. – *sensu lato*.

A total of 13 individuals failed to exhibit all character states of the most common types, and were assigned to the species that they mostly resemble – species in a broader sense (*sensu lato*, s.l.). Within the *Q. pubescens-Q. frainett*o group, the species were further discriminated following *Flora Europaea *descriptions [22].

**Figure 1:**
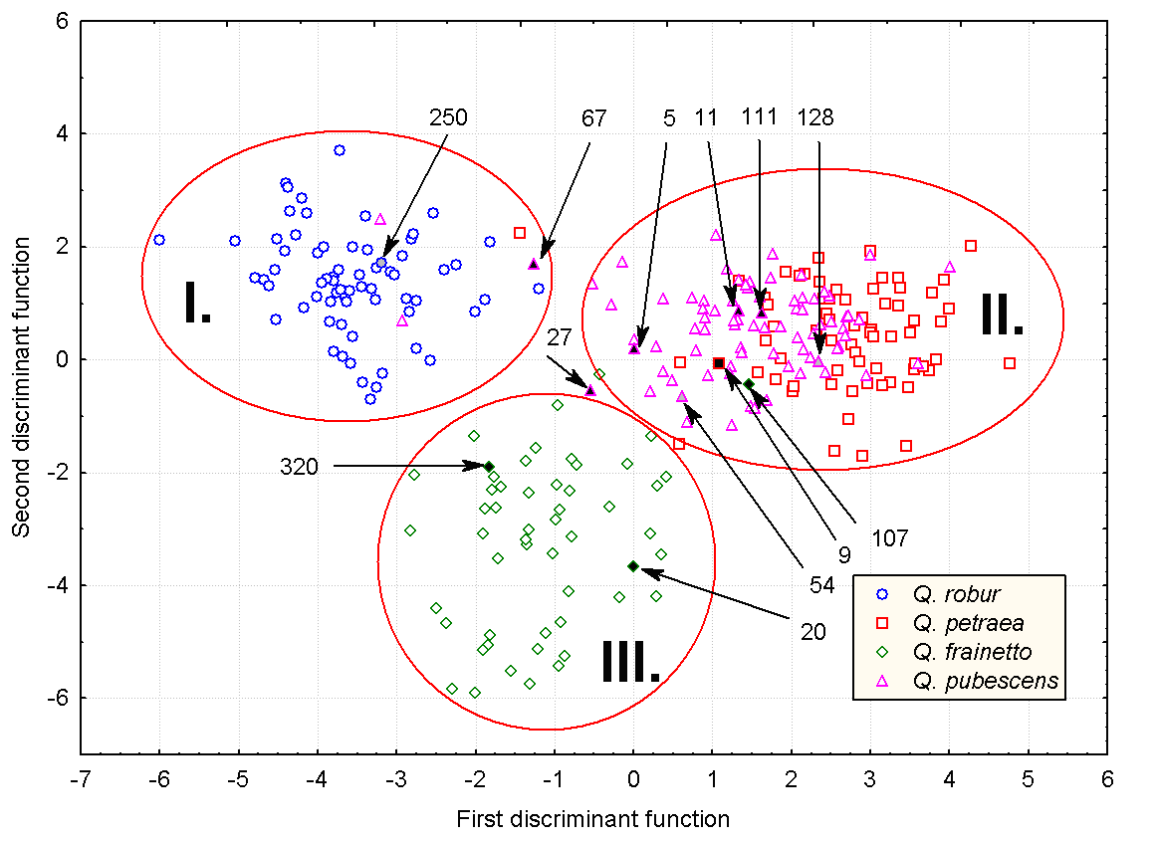
**Distribution of the individual scores for the first two discriminant functions using 13 leaf morphological traits**. The initial taxonomic classification of the individual trees (shape and colour of symbols) was based on pubescence characteristics (separation of *Q. robur*, *Q. petraea *and *Q. pubescens-Q. frainetto*), and species descriptions of *Flora Europaea *(separation of *Q. pubescens *from *Q. frainetto*). Nine putative first-generation hybrids and three misassigned individuals (54, 128 and 250) identified using the program STRUCTURE based on 13 genetic markers are shown by black and gray filled forms, respectively.

Each individual tree was classified to one of the four species based on the above mentioned procedure. Then we continued applying a multivariate approach (stepwise discriminant analysis) based on 13 leaf characters to test the first grouping of individuals to species. The discriminant analysis revealed three distinct groups (Figure 1). The first group consisted of all *Q. robur *s.s. individuals, two *Q. robur *s.l. individuals (68 and 200), three *Q. pubescens *s.l. individuals (24, 67 and 249) and one *Q. petraea *s.s. individual (64). The second group included *Q. petraea *and *Q. pubescens *individuals, but also two *Q. frainetto *individuals whereas the third group consisted of only *Q. frainetto *trees. The first and the second discriminant function accounted for 67.5% and 30.4% of the variation, respectively. Petiole ratio and basal shape of the lamina were the most important characters in distinguishing between species. As expected, only two species, *Q. petraea *and *Q. pubescens*, could not be discriminated on the basis of the leaf morphology alone. Moreover, there was no unambiguous separation between *Q. pubescens *and *Q. frainetto*, and consequently the assignment of trees showing intermediate discriminant scores may be subject to errors. In contrast, the separation between *Q. robur *and *Q. frainetto *was complete (Figure 1).

**Figure 2:**
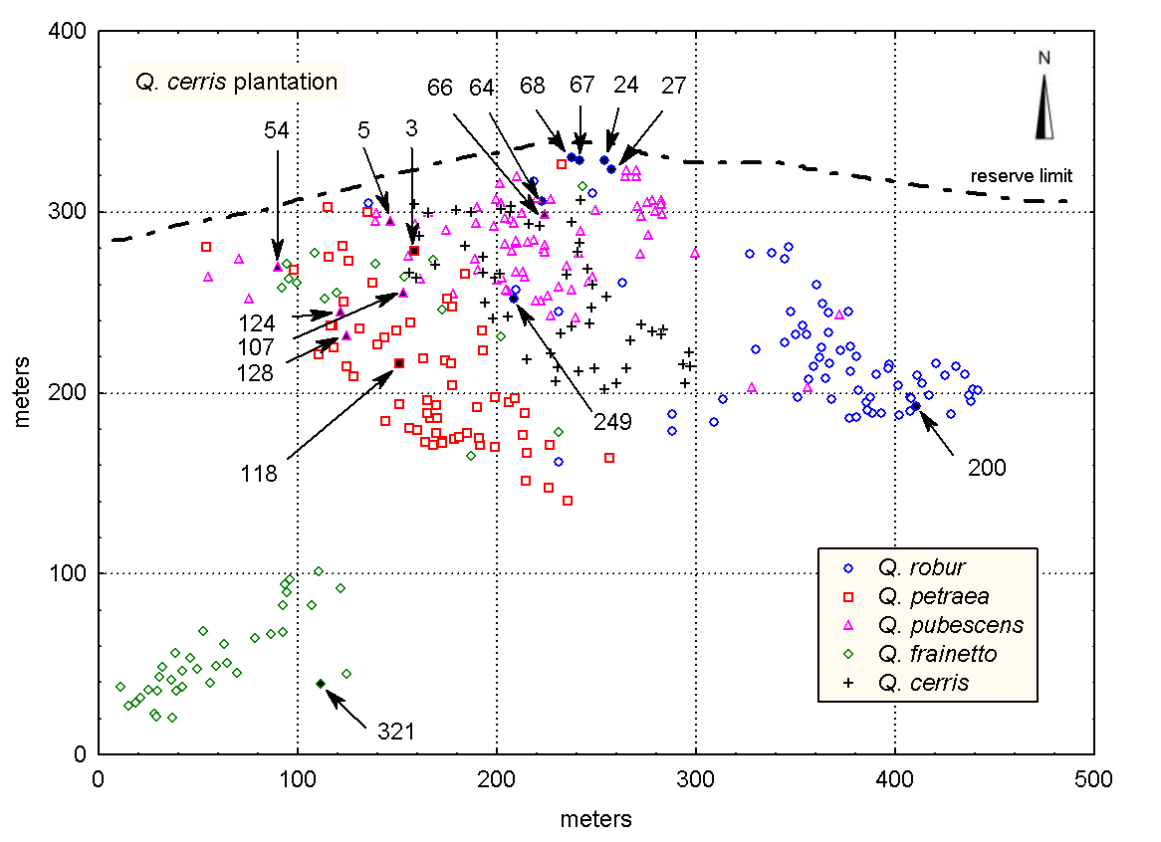
**Spatial distribution of phenotypically pure species and intermediate individuals in the study plot**. Each species is shown by a different shape and colour. Phenotypically intermediate individuals are indicated by arrows and filled forms. Shape and colour of symbols correspond to the species they mostly resemble.

In total, only six individuals were classified to other groups (species) by applying the discriminant analysis as compared to the grouping based on pubescence. Three out of these six individuals were categorized as '*sensu lato*' in the pubescence analysis. According to the assessment of pubescence and/or leaf morphology a total of 16 individuals (6.3%) revealed intermediate or contrasting character states between two species and were considered as a separate group in the further genetic analysis (Table 2). The number of unambiguously assigned (phenotypically pure) individuals to *Q. robur*, *Q. pubescens*, *Q. petraea *and *Q. frainetto*, was 65, 73, 65 and 50, respectively. Interestingly, most of the morphologically intermediate individuals were located in the contact zones between species rather than in pure stands of one species or another (Figure 2).

**Table 2:** Morphological and genetic assignment for individuals that exhibited intermediate characters or a combination of characters of different species.

Indv.	Morphological assignment		cpDNA haplotype	Genetic assignment			
			
	Pubescence	Leaf morphology		'robur'	'pubescens'	'petraea'	'frainetto'
68	*Q. robur *s.l.	*Q. robur*	6	0.16	0.81	0.02	0.01
24	*Q. pubescens *s.l.	*Q. robur*	5a	0.26	0.29	0.44	0.01
67	*Q. pubescens *s.l.	*Q. robur*	5a	0.54	0.43	0.02	0.01
249	*Q. pubescens *s.l.	*Q. robur*	5a	0.97	0.02	0.01	0.01
64	*Q. petraea *s.s.	*Q. robur*	5a	0.96	0.02	0.01	0.01
5	*Q. pubescens *s.l.	*Q. pubescens*	5a	0.88	0.08	0.03	0.01
27	*Q. pubescens *s.l.	*Q. pubescens*	5a	0.60	0.29	0.06	0.05
54	*Q. pubescens *s.l.	*Q. pubescens*	6	0.06	0.01	0.05	0.88
124	*Q. pubescens *s.l.	*Q. pubescens*	5a	0.04	0.18	0.75	0.04
128	*Q. pubescens *s.l.	*Q. pubescens*	5a	0.01	0.02	0.95	0.02
118	*Q. robur*-*Q. petraea *s.l.	*Q. petraea*	6	0.36	0.03	0.58	0.03
66	*Q. frainetto *s.s.	*Q. pubescens*	5a	0.01	0.96	0.01	0.03
107	*Q. frainetto *s.s.	*Q. pubescens*	5a	0.02	0.03	0.06	0.89
							
3	*Q. petraea *s.l.	*Q. petraea*	6	0.01	0.02	0.97	0.01
200	*Q. robur *s.l.	*Q. robur*	5a	0.96	0.01	0.01	0.02
321	*Q. frainetto *s.l.	*Q. frainetto*	5a	0.01	0.03	0.02	0.95

The morphological classification was based on pubescence and leaf morphology, respectively. The genetic assignment used a Bayesian method implemented in the program STRUCTURE. Individual's probability of belonging to each 'species' cluster is shown. s.s. – *sensu stricto*; s.l. – *sensu lato*.

Additionally, we applied the discriminant function proposed by [8] for distinguishing *Q. robur *from *Q. petraea*. This function relies on only two leaf characters: petiole length and number of intercalary veins. The species status was confirmed for all individuals from our *Q. robur *and *Q. petraea *sample, respectively. Moreover, as expected [8], most of the *Q. pubescens *trees (94.5%) were classified as *Q. petraea*.

### Genetic analysis

**Figure 3:**
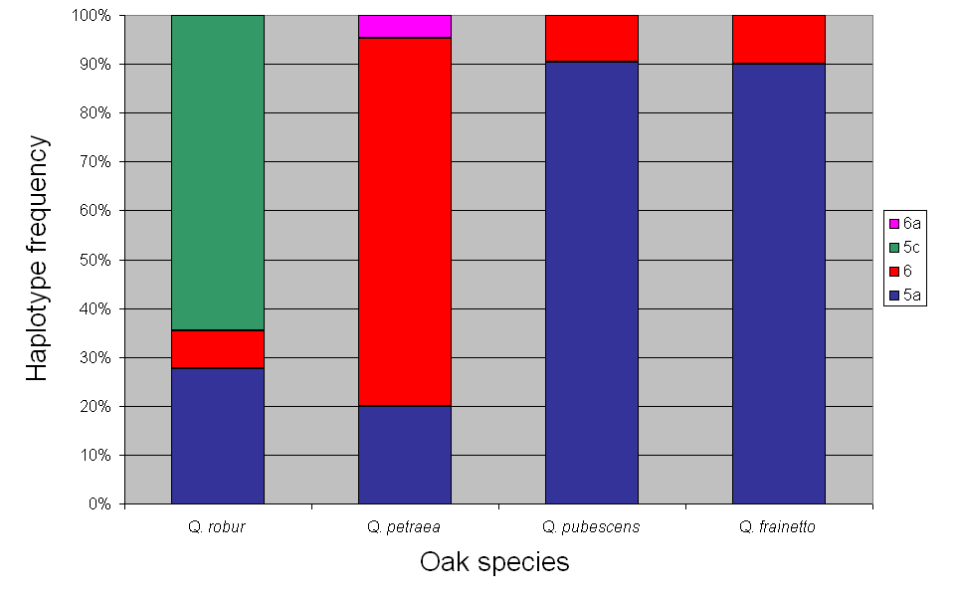
**Chloroplast DNA genetic structure**. Haplotype frequency and number is given for each phenotypically pure species.

A total of four chloroplast DNA haplotypes were found in the white oak complex. The most frequent haplotypes, 5a and 6, were identified in all species, although they were not evenly distributed among oak species (Figure 3). Haplotype 6 was predominant in *Q. petraea *and haplotype 5a in *Q. pubescens *and *Q. frainetto*, respectively. Interestingly, 42 (~65%) *Q. robur *individuals showed haplotype 5c, which was not found in the three other species. A very rare haplotype (6a) was confined to *Q. petraea*.

At microsatellite loci, among a total of 60 tests for linkage disequilibrium between pairs of loci only 6 were significant (P < 0.05). For isozymes, among 84 tests only in 5 cases the linkage disequilibrium was significant. Markers in significant linkage disequilibrium were only partly located on the same linkage group. Only 2 out of 24 tests for microsatellites (ssrQpZAG36 – *Q. robur*, P = 0.004; MSQ13 – *Q. pubescens*, P = 0.04) and 2 out of 28 tests for isozymes (Aap-A and Acp-C – *Q. petraea*, P = 0.03) showed significant deviations from genotypic frequencies expected under Hardy-Weinberg equilibrium [20]. Thus both categories of nuclear markers were considered to meet the assumptions for applying the Bayesian method implemented in the program STRUCTURE to assign individuals to species.

#### Assignment of individuals without any a priori information on morphology

**Figure 4:**
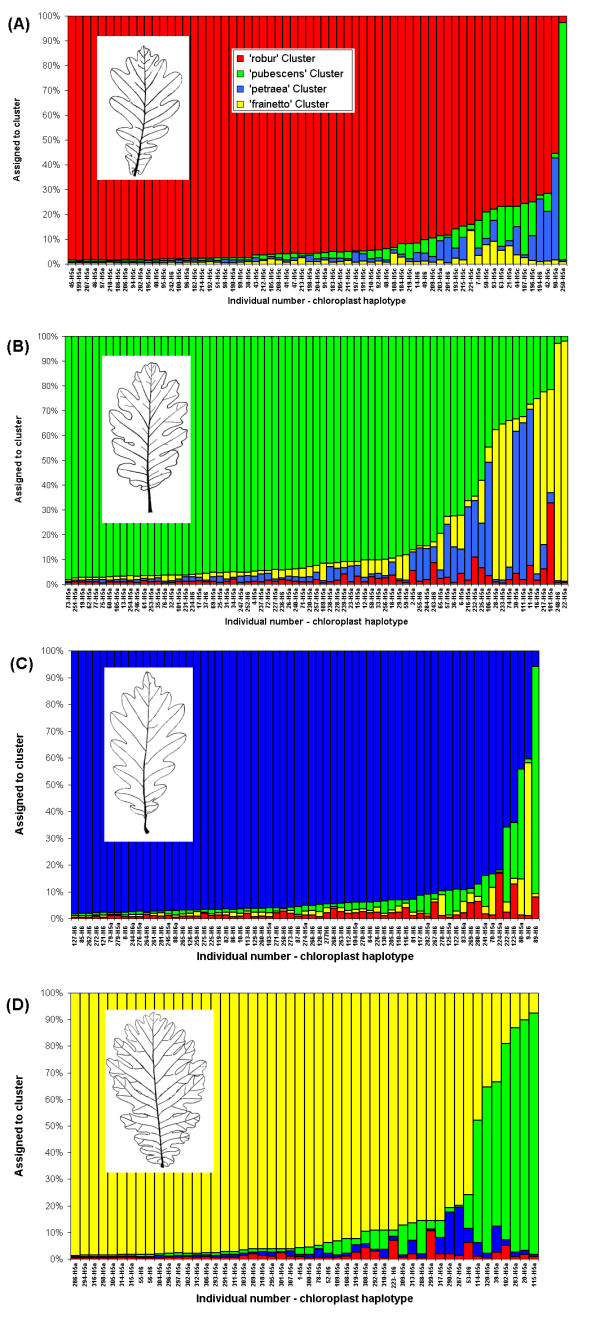
**Results of the genetic assignment based on the Bayesian method implemented in the program STRUCTURE**. Individuals are grouped according to their physical appearance – only the phenotypically pure species are shown: (A) – *Q. robur*, (B) – *Q. pubescens*, (C) – *Q. petraea*, (D) – *Q. frainetto*. Each individual is represented by a thin vertical line, which is partitioned into 4 coloured segments that represent the individual's probability of belonging to the cluster with that colour. Tree number and chloroplast DNA haplotype are given.

For the first modelling approach in the STRUCTURE program all individuals, morphologically pure species and intermediates were combined into one data set, without any *a priori *species assignment. Given *X*, the observed genotypes, the values of log likelihood of the multilocus genotype data, ln Pr(*X*|K), as a function of the number of clusters, K, were as follows: ln Pr(*X*|K) = -10868 for K = 1, ln Pr(*X*|K) = -10255 for K = 2, ln Pr(*X*|K) = -9851 for K = 3, ln Pr(*X*|K) = -9609 for K = 4, ln Pr(*X*|K) = -9635 for K = 5 and ln Pr(*X*|K) = -9683 for K = 6. The corresponding values for Pr(*X*|K) reach a maximum for K = 4. Consequently, the STRUCTURE program determined that four genetic clusters best fit the data in the species complex, which agrees with the existence of four morphological groups. There was a clear correspondence between the inferred genetic cluster and the species designation (Figure 4A–D and Additional file 1).

Within each phenotypically pure species an individual was considered to be assigned to the corresponding species cluster when it has an equal to or greater than 0.90 probability of belonging to that cluster. Introgressed forms are defined here as those showing less than 0.90 probability of belonging to their own species cluster and more than 0.10 probability of belonging to other species clusters. However, because our oak complex consists of four species and the amount of genetic information may be limited for some individuals, there were another two cases: 17 individuals, evenly distributed across species, showed less than 0.90 probability of belonging to their own species cluster and also less than 0.10 probability of belonging to other species clusters (e.g., individual 93, which was assigned to *Q. robur *showed 0.78, 0.08, 0.05 and 0.09 probability of belonging to 'robur', 'petraea', 'pubescens' and 'frainetto' cluster, respectively); 4 individuals showed less than 0.90 probability of belonging to their own species cluster and more than 0.10 probability of belonging to two other clusters (e.g., individual 181, which was assigned to *Q. pubescens *showed 0.19, 0.34, 0.04 and 0.43 probability of belonging to 'pubescens', 'robur', 'petraea' and 'frainetto' cluster, respectively). These individuals were considered neither assigned nor introgressed forms.

Following the above-mentioned classification scheme, the fraction of individuals assigned correctly to their species cluster varied among species, ranging from 80% for *Q. petraea *to 64% for *Q. frainetto*. The amount of introgression varied markedly between pairs of species, however, ranging from 1.7% (2 out of 115) between *Q. robur *and *Q. frainetto *to 16.2% (20 out of 123) between *Q. pubescens *and *Q. frainetto *(Figure 4A–D). The differences in the proportions of introgressive forms among species combinations are only partly explained by the mean geographic distance between individuals from different species. Noteworthy, the proportion of introgressed trees between *Q. frainetto *and *Q. pubescens *is not predictable from the mean geographic distance between the individuals of the two species.

Evidence of apparently asymmetrical gene flow was found between *Q. petraea *and *Q. robur *(1/6, the ratio between the number of introgressed forms with *Q. robur *detected among phenotypically pure *Q. petraea *and the number of introgressed forms with *Q. petraea *detected among phenotypically pure *Q. robur*) but also between *Q. pubescens *and *Q. robur *(0/6).

**Figure 5:**
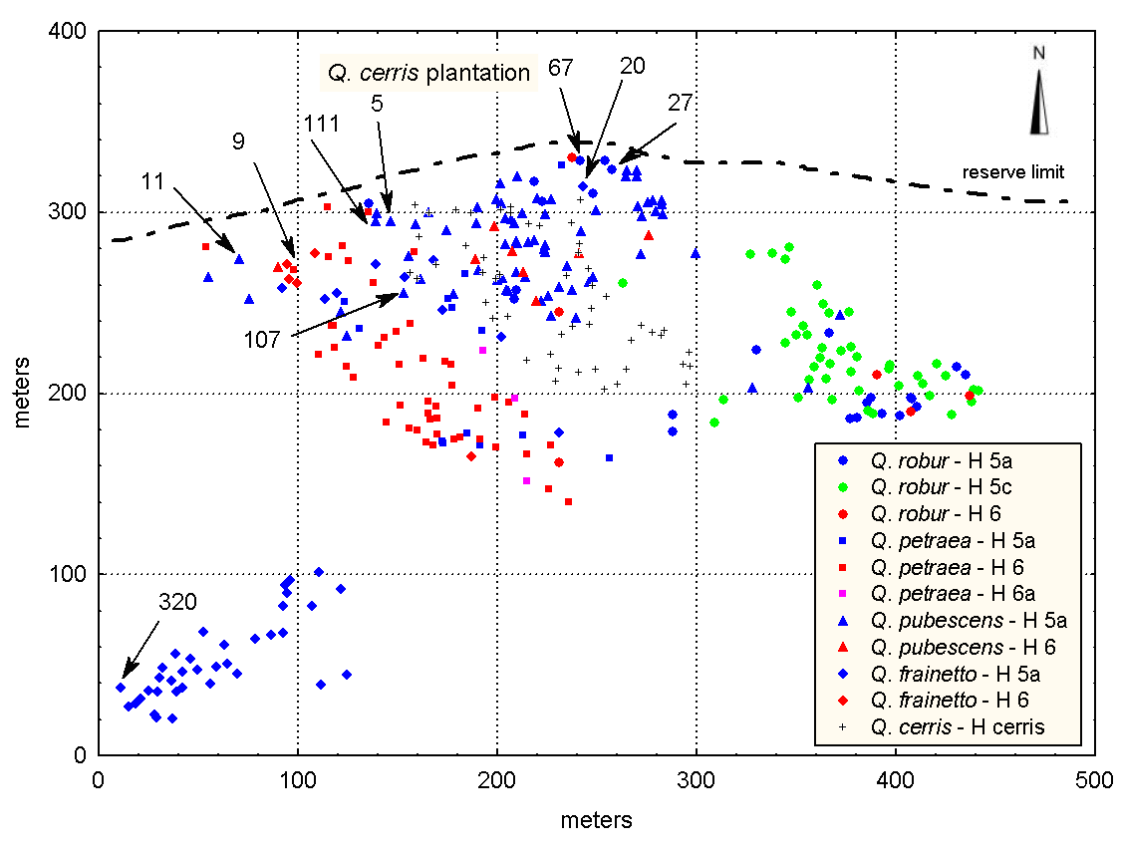
**Spatial distribution of oak species and chloroplast DNA haplotypes in the study plot**. Spatial positions of nine putative first-generation hybrids identified using a model-based Bayesian method implemented in the program STRUCTURE are indicated by arrows.

Furthermore, there seems to be no relationship between the chloroplast haplotype and the degree of admixture for each individual within each species (see Figure 4A–D). Only within *Q. robur*, the sub-group consisting of individuals with the chloroplast haplotype 5c, that is restricted to *Q. robur *at this site (Figure 5), showed a higher proportion of membership in the '*robur' *cluster (0.931) relative to the other sub-groups: *Q. robur *– H6 (0.887) and *Q. robur *– H5a (0.855). This observation may support the hypothesis that *Q. robur *individuals containing the haplotype 5c are the last 'immigrants' in the area and that there was less opportunity for them to hybridize with other species.

The results of the genetic assignment for individuals that belong to the morphologically intermediate class are shown in Table 2. The species designation based on leaf morphology as compared to pubescence showed a higher correlation with the genetic cluster indicated by STRUCTURE. However, there were only three instances of incongruent morphological and genetic marker discrimination.

#### Incorporating additional information in the assignment procedure

Next, we tested in STRUCTURE whether any individual in each species sample is misassigned, i.e. incongruence between morphology and molecular markers, or is a first-generation hybrid between species. For this purpose, we incorporated species information into the inference procedure. Each of the morphologically intermediate individuals was included into the species that it mostly resembled. According to this second approach the vast majority of individuals (95.5%) were assigned correctly, 9 individuals (3.4%) showed >0.50 probability of being first-generation hybrids and only 3 individuals (1.1%) were misassigned (*ν *= 0.10; Table 3). The probabilities of being F_1 _hybrids were highly consistent across different *ν *values in two instances (individual 9 and 320). However, the other individuals still have moderate to high probabilities of being first generation hybrids at *ν *= 0.01. *Q. pubescens *was identified as parental species in 8 out of 9 putative first generation hybrids. Interestingly, only 4 out of 9 putative F_1 _hybrids exhibited intermediate morphologies (Table 3). Accordingly, results from controlled crosses suggested that physical appearance of hybrid individuals often resembles the morphology of the female parent (e.g., [23]). Thus, nearly all putative F_1 _hybrids are located in areas of contact between the parental species and share the chloroplast haplotype with the neighbouring trees (Figure 5).

**Table 3:** Testing whether particular trees are misassigned or are first generation hybrids.

Tree number	Phenotypically assigned to species	ν^b^	No hybrid ancestry	Misassigned to species	F_1 _hybrid
				*Q. pubescens*	*Q. robur *× *Q. pubescens*
67^a^	*Q. robur*	*0.01*	0.55	0.00	0.43
		*0.05*	0.16	0.00	0.81
		*0.10*	0.06	0.00	0.91
				*Q. robur*	*Q. pubescens *× *Q. robur*
27^a^	*Q. pubescens*	*0.01*	0.85	0.00	0.15
		*0.05*	0.47	0.01	0.51
		*0.10*	0.25	0.02	0.72
				*Q. robur*	*Q. pubescens *× *Q. robur*
5^a^	*Q. pubescens*	*0.01*	0.08	0.57	0.35
		*0.05*	0.01	0.51	0.47
		*0.10*	0.00	0.48	0.52
				*Q. petraea*	*Q. pubescens *× *Q. petraea*
11	*Q. pubescens*	*0.01*	0.73	0.00	0.27
		*0.05*	0.29	0.01	0.71
		*0.10*	0.14	0.01	0.86
				*Q. petraea*	*Q. pubescens *× *Q. petraea*
111	*Q. pubescens*	*0.01*	0.83	0.01	0.17
		*0.05*	0.42	0.02	0.57
		*0.10*	0.21	0.02	0.77
				*Q. frainetto*	*Q. pubescens *× *Q. frainetto*
107^a^	*Q. pubescens*	*0.01*	0.74	0.07	0.19
		*0.05*	0.32	0.19	0.48
		*0.10*	0.16	0.26	0.58
				*Q. frainetto*	*Q. petraea *× *Q. frainetto*
9	*Q. petraea*	*0.01*	0.08	0.00	0.92
		*0.05*	0.02	0.00	0.98
		*0.10*	0.01	0.00	0.99
				*Q. pubescens*	*Q. frainetto *× *Q. pubescens*
20	*Q. frainetto*	*0.01*	0.70	0.04	0.26
		*0.05*	0.27	0.10	0.62
		*0.10*	0.14	0.12	0.72
				*Q. pubescens*	*Q. frainetto *× *Q. pubescens*
320	*Q. frainetto*	*0.01*	0.43	0.07	0.50
		*0.05*	0.08	0.14	0.78
		*0.10*	0.03	0.17	0.79
				*Q. pubescens*	*Q. robur *× *Q. pubescens*
250	*Q. robur*	*0.01*	0.81	0.11	0.08
		*0.05*	0.42	0.36	0.21
		*0.10*	0.20	0.54	0.26
				*Q. petraea*	*Q. pubescens *× *Q. petraea*
128^a^	*Q. pubescens*	*0.01*	0.08	0.67	0.25
		*0.05*	0.02	0.71	0.27
		*0.10*	0.01	0.75	0.25
				*Q. frainetto*	*Q. pubescens *× *Q. frainetto*
54^a^	*Q. pubescens*	*0.01*	0.18	0.62	0.21
		*0.05*	0.03	0.73	0.24
		*0.10*	0.01	0.75	0.24

^a ^individual previously included in the phenotypically intermediate class

^b ^probability of mixed ancestry

Values represent probabilities. Only the individuals showing a probability of being misassigned or first generation hybrid >0.50 at *ν *= 0.10 are shown.

The number of misassigned individuals was very small relative to the total sample size (3 out of 269). However, for individual no. 250 the probability of being misassigned to *Q. pubescens *is very sensitive to the choice of *ν*, which indicates that the amount of information in the genetic data is not sufficient to draw strong conclusions. The phenotype of this tree is typical for *Q. robur *(see Figure 1) ruling out any possibility of wrong morphological assignment. Individual no. 250 is the only member of the *Q. robur *population that does not possess any copy of the 'diagnostic' allele 136 bp at locus *ssrQpZag96 *[20] which may also affect its genetic assignment. For the two other misassigned individuals (128 and 54), the probability of belonging to *Q. petraea *and *Q. frainetto*, respectively, was highly consistent irrespective of the *ν *value (Table 3) showing a discrepancy between morphological and genetic assignment. Indeed, it seems that the criterion used for classifying individual 128 as *Q. pubescens*, fasciculate trichomes on the abaxial surface of the leaf, is not always specific for *Q. pubescens *as suggested by Aas [21]. Individual 54 showed a marginal position in the *Q. pubescens *group and was very close to *Q. frainetto *according to the results of the discriminant analysis (see Figure 1).

## Discussion

By using pubescence and leaf morphological characters we were able to distinguish between four closely related oak species in an area of sympatry. Only a small portion of individuals (~6%) could not be assigned unambiguously to one species or another and was categorized as morphologically intermediate. Many studies report a small number of individuals showing intermediate morphologies between *Q. robur *and *Q. petraea *(e.g., [8-10,24]). By contrast the morphological variation between *Q. pubescens *and *Q. petraea *was seldom studied and the results were contradictory. For example, a large proportion of morphologically intermediate forms between *Q. pubescens *and *Q. petraea *was detected in north-eastern France on the basis of thirty-four morphological traits [10] and in Switzerland based on observations of trichomes on leaves and twigs [25,26]. Other studies using pubescence and/or micromorphological characters differentiated very well *Q. petraea *from *Q. pubescens *in Central Europe and Italy [21,27]. Our results concerning the morphological differences between *Q. pubescens *and *Q. petraea *are consistent with the later studies. Reports of intermediate forms between *Q. robur *and *Q. pubescens *are very rare and rely on morphological observations [21]. We found several individuals that were morphologically intermediate between *Q. robur *and *Q. pubescens *and, indeed, some of them (e.g., individual 67) were putative genetic hybrids (Table 2). However, our observations suggest that apparently intermediate phenotypes between two species are not necessarily hybrids. Therefore, the inference of hybridization based on morphological characters, especially in oaks which possess a wide intraspecific variability, remains limited and can lead to wrong conclusions.

The presence of four differentiated morphological species was strongly supported by the genetic analysis that also identified the same number of genetic clusters. In addition, the majority of the individuals were assigned to the species cluster they were classified based on morphology (see Figure 4A–D). Our study provides no evidence for a breakdown of species pairs into a hybrid swarm and indicates the existence of reproductive barriers among species. However, the reproductive isolation is not complete, since a substantial number of genetically intermediate individuals was detected within each phenotypically pure species. Interspecific gene flow and/or recent divergence of the species with retention of ancestral polymorphism (e.g., [19]) may explain the occurrence of these individuals. However, the spatial locations of nearly all putative F_1 _hybrids in contact zones between species (Figure 5) points to interspecific hybridization as origin of genetically intermediate individuals. Even for one *Q. frainetto *individual (no. 320), a putative F_1 _hybrid between *Q. frainetto *and *Q. pubescens*, which appears to be located at a considerable spatial distance of any *Q. pubescens *tree (Figure 5), several *Q. pubescens *trees were observed in its south-western vicinity (location not shown in Figure 5). The predominant location of introgressed forms in contact areas between species has been reported in other studies on oak hybridization [11,28]. Contact areas might represent ecotones for edaphic and hydrological parameters being more favourable for the establishment of hybrids (e.g., [29]). At Bejan, each oak species occupies a different ecological niche and environmental variation is mentioned across the site [30]. It was suggested that the success of an hybridization event in *Quercus *strongly depends on the habitat conditions [5].

The fraction of putative F_1 _hybrids was very low in the present study – 3.4% (9 out of 269). Only four of them were previously classified as morphologically intermediates. Similar results were found in a mixed stand of *Quercus lobata *and *Q. douglasii *in North America: only one of the three individuals that showed the highest probability of hybrid ancestry (0.20–0.25) was intermediate in appearance [31]. Similarly, a very small fraction (2%) of potentially F_1 _hybrids (those having a posterior probability of 40–60%) between *Q. petraea *and *Q. pyrenaica *were identified in a mixed stand in central Spain [28], but in the absence of any morphologically intermediate tree.

Interestingly, the asymmetric introgressive gene flow from *Q. petraea *to *Q. robur *is consistent with the model of asymmetric hybridization between the two species [15]. Both species flower in synchrony when they cohabit in mixed stands (e.g., [24]) and evidence of long distance pollen transport was found [17]. Controlled crosses have also demonstrated that *Q. robur *and *Q. petraea *are compatible and most of the hybrids were from the combination *Q. robur *(female) × *Q. petraea *(male) indicating a lower success of *Q. robur *as pollen donor (e.g., [32,33]). The rate of introgression between the two species was estimated at 6.1% which is relatively similar to the level of interspecific gene flow (7.5%) detected by paternity analysis in a mixed stand [17].

This study provides no evidence of extensive hybridization between *Q. robur *and *Q. frainetto*. First, no morphologically intermediate tree between the two species was observed at Bejan. Second, the estimated rate of introgression was the lowest among all pairs of species 1.7% (2 out of 115). Our finding contradicts a phylogenetic study based on ITS sequences in 12 Italian oak taxa that showed a closer affinity between the two species as compared to *Q. petraea *and *Q. pubescens *[34]. However, spatial arrangement of trees belonging to both species (Figure 1) and the low density of *Q. frainetto *in the whole reserve may provide little opportunity for interspecific gene flow between these two species. On the other hand, the study of Italian oaks is based on only one individual sample from *Q. robur *and *Q. frainetto*, respectively.

The highest amount of introgression 16.2% (20 out of 123) was observed between *Q. frainetto *and *Q. pubescens*. This result is consistent with the chloroplast sharing between the two species (Figure 3). According to Schwarz's taxonomical scheme [35] both species are grouped in section *Dascia *which may indicate a closer affinity between *Q. pubescens *and *Q. frainetto *and a higher propensity for hybridization relative to other species. However, since the two species are not unambiguously separated by morphological characters (see Figure 1), a wrong *a priori *assignment of individuals to species may bias the estimation of hybridization between the two species (see for example the case of individual 54 in Table 2). The introgression between *Q. pubescens *and *Q. petraea *was relatively high ~9.4% (13 out of 138) which is consistent with the low levels of genetic differentiation observed between the two species in Italy [27] and at Bejan forest [20]. Furthermore, pollination experiments revealed that hybridization between *Q. pubescens *on the one hand and *Q. robur *and *Q. petraea *on the other hand is possible [25].

An unambiguous genetic assessment of hybridization rates within a species complex would require highly discriminatory markers [e.g., [36]]. Such markers are very rare in the two white oak species, *Q. robur *and *Q. petraea *[[19,37] and references therein]. The differences between the two species derive mostly from gradations in allele frequencies rather than from distinctive genotypes. Even though the nuclear genetic differentiation between *Q. robur *and *Q. petraea *is low, the two species remain separate genetic entities across their natural range [38,39]. Considering the low linkage disequilibrium between marker pairs and their wide genomic distribution [37,40] even the limited number of nuclear markers (seven isozymes and six microsatellites) is expected to reflect genome wide differentiation patterns among the four investigated species.

Predominantly maternal effects, i.e. hybrids are more similar to the species of their maternal parent, may explain the presence of introgressed forms amongst the phenotypically pure species [8,24]. It has been observed in controlled crosses between *Q. robur *and *Q. petraea *that first generation hybrids at juvenile stage exhibited a leaf morphology that resembles the morphology of the female parent very much [23]. However, it is unclear whether these maternal effects persist at a later stage.

The maintenance of four distinct gene pools in an area of sympatry may be explained by selection against hybrids (e.g., [8,15]). The local site conditions would be more favourable to the parental species rather than to the F_1_hybrids, that are gradually eliminated until the adult stage is reached (disruptive selection). Furthermore, the small number of first generation hybrids compared to the high number of potentially introgressed forms also suggests a lower F_1 _hybrid viability relative to other hybrid classes. Those hybrids resulting from backcrossing are more parental-like and would be better able than F_1 _hybrids to live under optimal environmental conditions for one species or another. Our results are consistent with other studies reporting a low fraction of first generation hybrids relative to later generation hybrids in natural populations (e.g., [41]).

Differences in flowering phenology between species can be a serious constraint for interspecific gene flow and may account for the pattern of hybridization detected in this species complex. Phenology was not directly assessed in the study area, but observations on flowering time had been carried out in several mixed stands consisting of *Q. robur *and *Q. petraea *from the same geographical region [42]. The data revealed a partial overlap of the flowering period, with *Q. robur *starting flowering earlier than *Q. petraea*. Unfortunately, phenological observations are missing for *Q. pubescens *and *Q. frainetto*, respectively.

## Conclusion

The present study has documented the occurrence of historical gene flow within a species-rich natural community of oaks. There was a very good correspondence of each morphological species with one of the inferred genetic clusters. The amount of gene flow between species appears to be at relatively low rates and does not represent a '*threat' *for the species identity which has been maintained in this area of sympatry. Genotyping of progenies using highly variable microsatellite markers for paternity analyses will provide information about the ongoing level of gene flow between species. This will also enable us to determine whether there is a decrease in hybrids' frequency between the seed and adult stage, due to selection events. Moreover, collecting seeds and raising seedlings from the putative first generation hybrids would allow investigating the segregation of the morphological traits involved in species differentiation.

## Methods

### Study area and plant material

Trees were sampled at Bejan Forest (45°51'N, 22°53'E), an oak reserve situated at the foothill of the Carpathian mountains in west-central Romania. The reserve is located on a south-east facing hillside at an elevation of 250–380 m above sea level and experiences a continental climate with Atlantic and sub-Mediterranean influences. Five oak species in a broad sense (*sensu lato*) are common at Bejan: four closely related species (known also as white oaks) – *Q. robur*, *Q. petraea*, *Q. pubescens *and *Q. frainetto*, respectively – all belonging to section *Quercus *sensu stricto (s.s.) and *Q. cerris *from section *Quercus *s.l. or "*Cerris *group" [43,44]. Other two 'species', *Q. dalechampii *and *Q. virgiliana*, were also mentioned to occur in the reserve [30], but these taxa are not easily distinguishable and their taxonomic ranking remains uncertain. Currently, the two taxa are included in *Q. petraea *and *Q. pubescens*, respectively (e.g., [45]). *Q. cerris *is not considered in this study since no evidence of hybridization between *Q. cerris *and the other four oak species was found so far [46,47].

A study plot was established in the species-richest part of the reserve, which shows a pronounced environmental variability [30]. Here, the oak species cohabit along a gradient of water and nutrient availability. *Q. robur *grows on nutrient-rich and wetter soils in the eastern part of the reserve at the bottom of the slope (Figure 2). In contrast, *Q. petraea *prefers the more acidic and better drained soil in the south-western part of the study plot and is also predominant in the rest of the nearby stands. *Q. pubescens *grows on a sunny and relatively dry slope in the upper part of the hillside, at the highest altitude. *Q. frainetto*, which is an element of the (sub-) Mediterranean flora, tolerates a heavy soil and is pretty well-adapted to xeric conditions.

A total of 269 white oak trees were mapped and sampled. The sampling was exhaustive within an area of approximately 4.5 ha (the core-plot), with no *a priori *selection of trees. Since *Q. frainetto *was less abundant within the core plot, we extended the sampling for this species to the nearby area outside of the core plot (Figure 2).

### Morphological data

During the summer 2004, three to five (on average 4.2) leaves and at least one current year shoot were sampled within the upper crown of each tree. Leaves and shoots were stored in a herbarium for further morphological analysis. We applied two procedures: the first one was previously used for the separation of *Q. robur*, *Q. petraea *and *Q. pubescens *in Central Europe on the basis of pubescence alone [21]; the second approach considers leaf morphological traits and was used by [8] for distinguishing *Q. robur *from *Q. petraea*. Pubescence was assessed with a stereomicroscope (×40) according to the procedure described by [21]. Six characters were considered: stellate and fasciculate hairs on the abaxial surface of the leaves, fasciculate hairs along and on the mid-rib, on the petiole and on the twig, respectively. Each character was scored as 1 (present) or 0 (absent) in order to determine different combinations of character states which are taxa specific [21]. Secondly, a suite of leaf morphological traits associated with differences between *Q. robur *and *Q. petraea *[8] was assessed in all individuals. These traits are dimensional (lamina length, petiole length, lobe width, sinus width, and length of lamina at the largest width); counted (number of lobes and number of intercalary veins); observed (basal shape of the lamina) and transformed characters (lamina shape, petiole ratio, lobe depth ratio, percentage venation and lobe width ratio). Based on these characters a multivariate approach (discriminant analysis) was carried out using the computer software STATISTICA version 7.1. [48].

### Genetic data

Chloroplast DNA, isozyme and microsatellite data used in this study are described in detail elsewhere [20]. Nomenclature of the chloroplast variants follows that used in European-wide inventories [13,46]. The seven enzyme loci used were: acid phosphatase (*Acp-C*), alanine-aminopeptidase (*Aap-A*), aspartate aminotransferase (*Aat-B*), isocitrate dehydrogenase (*Idh-B*), menadione-reductase (*Mnr-A*), 6-phosphogluconate-dehydrogenase (*6-Pgdh-B*), and phosphoglucose-isomerase (*Pgi-B*). The electrophoretic procedures and the verification of Mendelian inheritance are given elsewhere [49,50]. Six nuclear microsatellite loci, linkage group in brackets (after [40,51]) were analysed: *ssrQpZAG1*/5 (7), *ssrQpZAG9 *(7), *ssrQpZAG36 *(2) and *ssrQpZAG104 *(2) were developed for *Q. petraea *[52], *ssrQrZAG96 *(10) was developed for *Q. robur *[53] and *MSQ13 *(6) was developed for *Q. macrocarpa *[54], respectively. Details of the methods used for DNA extraction, PCR amplifications, and microsatellite genotyping are given in a companion paper [20].

### Genetic assignment

We used the Bayesian model-based clustering method implemented in the program STRUCTURE version 2.1 [55] to assign individuals to K populations (species in our case) on the basis of individual multilocus genotypes. This allowed us to analyze the correspondence between the morphologically based species and inferred genetic structure. We conducted a series of independent runs of the Gibbs sampler for each value of K (the number of species) between 1 and 6. The results presented here are based on runs of 10^6 ^iterations, following an initial burn-in period of 30,000 iterations. Performing a series of trial runs we found that using these parameters we obtained consistent estimates of posterior probabilities of K. The program was run without any information regarding species identification (USEPOPINFO = 0) and in the admixture mode in which the fraction of ancestry from each cluster is estimated for each individual. We used the correlated allele frequency model, which often improves clustering for closely related populations (species), but may increase the risk of over-estimating the number of clusters [56]. We thus continued our analysis to explore how well this structure corresponded to our morphological assignment of individuals to species and to detect putative hybrids, using prior information (USEPOPINFO = 1). We ran the analysis at different values of 'immigration' rate *ν *('hybridization' in our context), as recommended [55], in order to evaluate the robustness of results to the choice of *ν*. We performed analyses for *ν *= 0.01, 0.05, and 0.10, which are considered as estimates for hybridization within the European white oak complex, based on data from paternity analysis at the same site (Curtu et al. unpublished data) and on data in the literature [17]. We report the posterior probabilities that the individual in question is correctly assigned to the given species, or has ancestry in the other species. The Bayesian clustering method requires that the marker-loci are unlinked and at linkage equilibrium with one another within populations [55]. Therefore, to verify the independence of our marker loci we analysed linkage disequilibrium for all pairs of loci in each species sample with exact tests. The program GENEPOP version 3.4 [57] was used for the computations.

To further test the morphological classification we conducted an analysis in GENECLASS2 [58]. We used a distance-based (Cavalli-Sforza and Edwards chord distance) assignment method, which in contrast to the previous method has the advantage of not assuming Hardy-Weinberg equilibrium or absence of linkage disequilibrium among loci (e.g., [59]). Since the results of this assignment procedure were highly consistent with those generated using the Bayesian method (data not shown), only STRUCTURE results are presented here.

In order to test whether the level of introgression between species is simply a result of the spatial configuration of oak species at this site, mean geographic distances between species were calculated. GenAlEx6 software [60] was used for calculating geographic distances between individuals of the different species.

## Authors' contributions

ALC conceived the idea, collected the samples, analyzed the morphological and genetic data, and wrote the majority of the text. OG participated in the molecular genetic study, helped to analyze the data and to draft the manuscript. RF participated in the design and coordination of the study, data interpretation and made substantial contributions to writing the paper. All authors read and approved the final manuscript.

## Supplementary Material

Additional file 1 **Inferred ancestry and probability intervals**. EXL file giving detailed information about inferred ancestry and probability intervals for each sample. A probability interval is the Bayesian analogue of a confidence interval. Click here for file
